# Hospitalized cancer patients with comorbidities and low lymphocyte counts had poor clinical outcomes to immune checkpoint inhibitors

**DOI:** 10.3389/fonc.2022.980181

**Published:** 2022-09-14

**Authors:** Richard Benjamin Young, Hemali Panchal, Weijie Ma, Shuai Chen, Aaron Steele, Andrea Iannucci, Tianhong Li

**Affiliations:** ^1^ Division of Hematology/Oncology, Department of Internal Medicine, University of California Davis School of Medicine, University of California Davis Comprehensive Cancer Center, Sacramento, CA, United States; ^2^ Division of Biostatistics, Department of Public Health Sciences, University of California, Davis, Davis, CA, United States; ^3^ Department of Pharmacy Services, University of California (UC) Davis Health, University of California (UC) Davis Comprehensive Cancer Center, Sacramento, CA, United States

**Keywords:** immune checkpoint inhibitor (ICI), inpatient, survival outcome, comorbidities, absolute lymphocyte count (ALC), derived neutrophil to lymphocyte ratio (dNLR), hospitalized adult patients

## Abstract

**Background:**

Immune checkpoint inhibitor (ICI) therapy has improved survivals with a favorable toxicity profile in a variety of cancer patients. We hypothesized that hospitalized cancer patients who have acute or chronic comorbidities may have suppressed immune systems and poor clinical outcomes to ICIs. The objective of this study was to explore clinical outcomes and predictive factors of hospitalized cancer patients who received ICI therapy at an NCI-designated Comprehensive Cancer Center.

**Methods:**

A retrospective review of electronic medical records was conducted for adult cancer patients who received an FDA-approved ICI during admission from 08/2016 to 01/2022. For each patient we extracted demographics, cancer histology, comorbidities, reasons for hospitalization, ICI administered, time from treatment to discharge, time from treatment to progression or death, and complete blood counts. Progression-free survival (PFS) and overall survival (OS) were estimated using the Kaplan–Meier method and compared using the log-rank test. The 95% confidence interval for survival was calculated using the exact binomial distribution. Statistical significance was defined as 2-sided *p*<0.05.

**Results:**

Of 37 patients identified, 2 were excluded due to lack of complete blood counts on admission. Average hospital stay was 24.2 (95% CI 16.5, 31.9) days. Ten (27.0%) patients died during the same hospitalization as treatment. Of those who followed up, 22 (59.5%) died within 90 days of inpatient therapy. The median PFS was 0.86 (95% CI 0.43, 1.74) months and median OS was 1.55 (95% CI 0.76, 3.72) months. Patients with ≥3 comorbidities had poorer PFS (2.4 vs. 0.4 months; p=0.0029) and OS (5.5 vs. 0.6 months; *p*=0.0006). Pre-treatment absolute lymphocyte counts (ALC) <600 cells/µL were associated with poor PFS (0.33 vs. 1.35 months; *p*=0.0053) and poor OS (0.33 vs. 2.34 months; *p*=0.0236). Pre-treatment derived neutrophil to lymphocyte ratio (dNLR) <4 was associated with good median PFS (1.6 vs. 0.4 months; p=0.0157) and OS (2.8 vs. 0.9 months; p=0.0375).

**Conclusions:**

Administration of ICI therapy was associated with poor clinical outcomes and high rates of both inpatient mortality and 90-day mortality after inpatient ICI therapy. The presence of ≥3 comorbidities, ALC <600/μL, or dNLR >4 in hospitalized patients was associated with poor survival outcomes.

## Introduction

The advent of immune checkpoint inhibitor (ICI) therapy has revolutionized cancer treatment and improved survival outcomes for a variety of cancers globally (1–4). Since the deployment of these agents in 2011, the field of cancer therapy has witnessed an ever-expanding landscape of biomarker-driven precision oncology and novel treatments (5–7). Because of promising outcome data and favorable toxicity profiles, ICI has increasingly been integrated into the treatment of a variety of cancer types across all clinical settings. However, ICIs only work in subsets of cancer patients for each cancer type and can be associated with severe or even fatal immune related adverse effects (irAEs) (8). In the current era of precision oncology, it is critical to select the appropriate cancer patients who are most likely to benefit from ICI therapy (9). Historically, the focus of much research has been on the predicted value of PD-L1 expression in tissue and host biomarkers as a means to determine clinical response to ICI therapy (10–12). Currently, there are limited studies focused on understanding the impact of clinical factors on patient selection for ICI treatment, and the choice of whom to treat can sometimes represent a difficult question (13). For patients receiving chemotherapy, the presence of poor performance status (PS) and/or concurrent high comorbid burden are associated with low rates of disease control, progression-free survival (PFS) and overall survival (OS) in cancer populations (14). Performance status (ECOG) of >2 (15, 16), active autoimmune diseases (17), and concurrent use of high dose steroids (17, 18) have been associated with poor clinical response and/or high irAEs to ICI therapy.

In hospitalized cancer patients, ICI treatment is often deferred due to the uncovered cost and efficacy in this population is unclear. Intuitively, hospitalized cancer patients often have worse performance status (PS) and in the elderly these functional losses can often be irreversible (19). In patients with solid tumors and poor PS, inpatient chemotherapy is associated with high mortality (14). However, little is known for the clinical outcomes in hospitalized cancer patients with high comorbid burden who receive ICI treatment (20). ICI therapy sometimes represents the last treatment option for patients with advanced solid tumors. Our study explored the clinical factors and predictive biomarkers that can be used to select cancer patients who may derive long term clinical benefit from ICI treatment during admission at an NCI designated comprehensive cancer center.

## Materials and methods

We conducted a retrospective review of all adult (≥18 years old) cancer patients who received a FDA-approved ICI, either alone or in combination with chemotherapy, while admitted to inpatient services from August of 2016 through January of 2022 (i.e., 5 years) through a pharmacy database under an Institutional Review Board (IRB) approval protocol (University of California, Davis Protocol No. 937274). The ICIs used included ipilimumab, nivolumab, pembrolizumab, atezolizumab, durvalumab, avelumab and cemiplimab. Anonymized data were extracted from electronic medical records for age, gender, ethnicity, cancer histology, comorbidities, reasons for hospitalization, ICI administered, time from treatment to discharge, complete blood counts with differential, and clinical response to ICI treatment. Cancer types were classified by site of origin and defined as lung, melanoma, lymphoma, genitourinary, and “other.” Tumor histology, metastatic status, and date of diagnosis were obtained from outpatient records when applicable. Performance status (PS) assessment using the Eastern Cooperative Oncology Group (ECOG) criteria was provided by the treating oncologist (21). Comorbid burden was captured for each patient based on inpatient and outpatient documentation and evaluated by the Charlson Comorbidity Index (22, 23). Comorbid conditions that were evaluated in our study included evidence of any major organ failure (including heart, lung, kidney, and liver), thromboembolic disease (including pulmonary embolism or deep venous thrombosis), stroke, infection during inpatient stay requiring use of intravenous antibiotics, and malnutrition or failure to thrive recorded from either hospital notes (including history and physician notes, discharge summaries and inpatient progress notes) or from the problem list observed in the electronic medical record (EMR). The number of prior lines of therapy, type of therapy, and reason for treatment discontinuation were also recorded. Additionally, information on length of stay (LOS), time from treatment to discharge, time from ICI treatment to progression (PFS) or death (OS), absolute lymphocyte count (ALC) <600 cells/µL, derived neutrophil to lymphocyte ratio (dNLR) ≥4 prior to therapy on admission were computed and analyzed. Charlson Comorbidity Indices were independently calculated by two investigators (HP and RBY). Indications for ICI use were verified independently by at least two investigators (HP, RBY and TL). ICI expenditure data was calculated using wholesale average cost (WAC) which is the acquisition cost paid for drugs administered in the inpatient setting (AI). Equivalent data was obtained for the same medication administration in the outpatient setting using 340b costs. When relevant, information regarding immune-related adverse events (irAEs) was procured *via* review of inpatient progress notes, discharge summaries, and follow up oncology clinic notes. Last known follow-up and date of death were established by EMR review through January 26, 2022.

Data were summarized according to frequency and percentage for qualitative variables, and by mean ± standard deviation, median, and range for quantitative variables. PFS and OS were estimated using the Kaplan–Meier method along with their medians and relevant confidence intervals and compared using the log-rank test between groups. Cox proportional hazards models were used to estimate hazard ratios (HRs). Statistical significance was defined as 2-sided *p*<0.05.

## Results

### Patient characteristics

**Figure 1:**
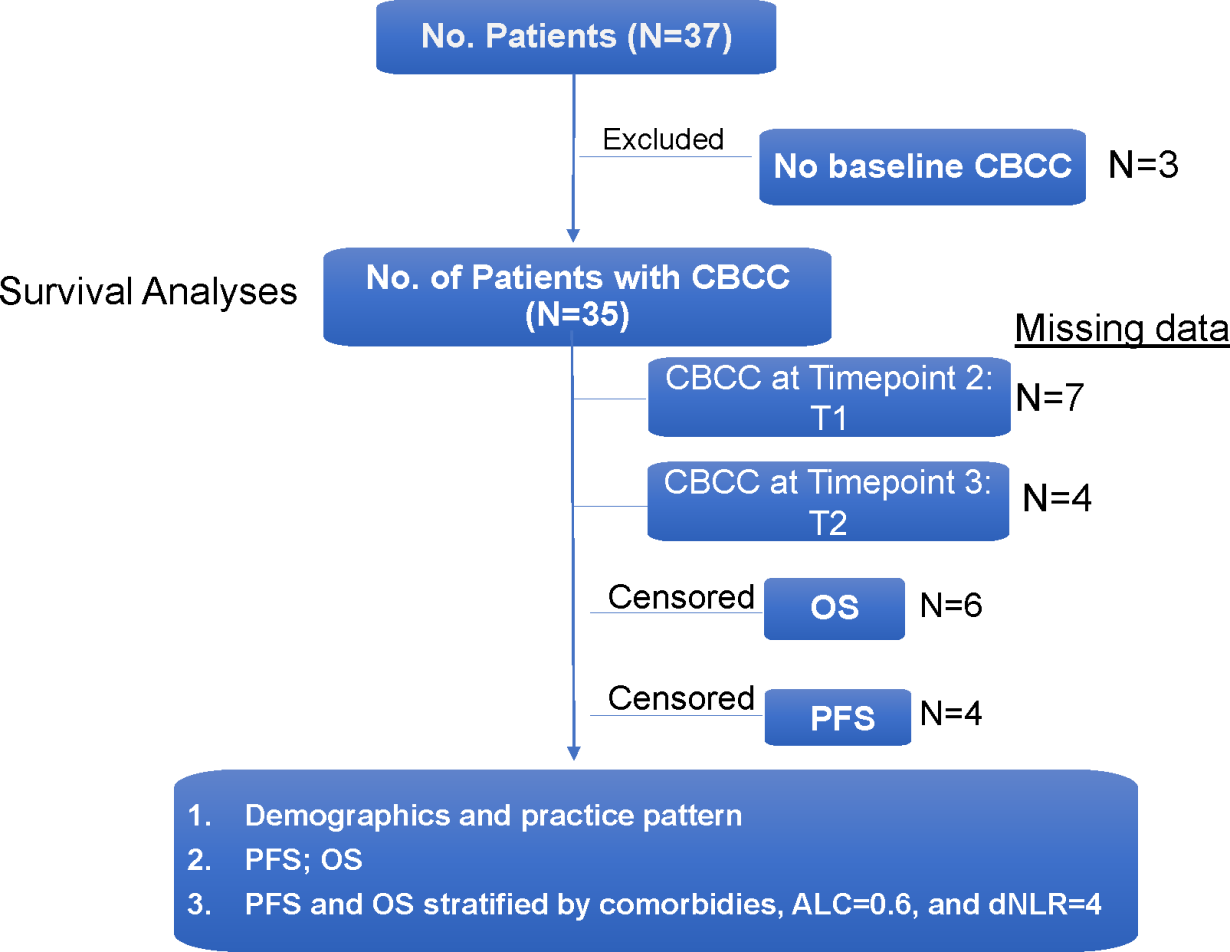
Summary of Study Patients.

Between August 1, 2016 and January 26, 2022, 37 cancer patients who received ICI therapy while admitted to the hospital were identified through the institutional pharmacy database (**Figure 1**). The majority of patients were male (78.4%) and of Caucasian descent (59.4%). The median age was 53.5 years with a range of 21 to 79 years of age. All patients had the FDA-approved indications to receive an ICI which is usually given in the outpatient setting. The known information of companion and complemental biomarkers for ICI is provided in Column H in **Supplemental Table 1**. Lung cancer was the most common cancer type (13, 35.1%) to receive inpatient ICI therapy, followed by melanoma (8, 21.6%), genitourinary (8, 21.6%), and lymphoma (4, 10.8%). Reasons for admission were variable, but frequently included infection (5, 13.5%) and initiation of cancer-directed therapy (14, 37.8%). Indications for inpatient ICI use were initiation of new treatment (13, 35.1%), emergent use for tumor progression (10, 27.0%), need for therapy while awaiting disposition (6, 16.2%), and convenience to the patient (6, 16.2%). Full patient demographic information is summarized in **Table 1**.

**Table 1:** Hospitalized patient demographics and characteristics.

No. Patients: N (%)	N=37
Male	29 (78.4%)
Female	8 (21.6%)
**Age: mean (range), yo**	53.5 (21-79)
**Race/Ethnicity:**	
White	22 (59.4%)
African American	5 (13.5%)
Hispanic or Latino	3 (8.1%)
Other	7 (18.5%)
**Type of cancer:**	
Lung	13 (35.1%)
Melanoma	8 (21.6%)
Lymphoma	4 (10.8%)
Genitourinary	8 (21.6%)
Others	4 (10.8%)
**Reason for admission:**	
Infection	5 (13.5%)
Initiate treatment	14 (37.8%)
Other	18 (48.6%)
**Reason for inpatient therapy:**	
Convenience to patient	6 (16.2%)
Assist with hospital disposition	6 (16.2%)
Initiation of new treatment	13 (35.1%)
Emergent for tumor progression	10 (27%)
Other	2 (5.4%%)

### Hospitalized cancer patients had poor clinical outcomes to ICI therapy

**Figure 2:**
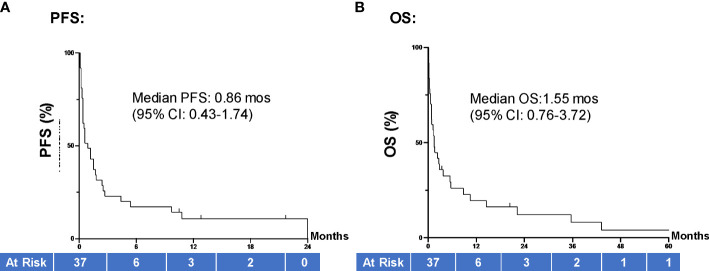
PFS and OS of all hospitalized cancer pts. **(A)** Median PFS for all patients in our study (N=37) was 0.86 months (95% CI 0.43, 1.74) and **(B)** median OS was 1.55 (95% CI 0.76, 3.72) months. Groups were compared using the log-rank test. Tick marks indicate censored data. P<0.05 indicates statistical significance. PFS, progression free survival; OS, overall survival.

The average length of hospital stays was 24.2 (95% CI 16.5, 31.9) days. With a median (range) follow-up of 1.3 (0.1-60.4) months, most patients died during the study period, and only 5 (13.5%) were alive at the time of data analysis. On review of both inpatient and outpatient records, the average number of comorbid conditions present in our population was 2.24. Charlson Comorbidity Index ranged from 2-18 points, with an average score of 8.5 points. The majority of patient ECOG functional assessments were rated by the treating oncologist as 1 (48.6%) or 2 (21.6%). Ten cases (27.0%) had an ECOG score of 3 and 1 case (2.7%) had an ECOG score of 4. Of all study patients, 10 (27.0%) patients died during the same hospitalization they received treatment. Amongst the patients who had follow-up data, 22 died within 90 days of inpatient ICI therapy. For the entire patient population, the median PFS was 0.86 (95% CI 0.43, 1.74) months and median OS was 1.55 (95% CI 0.76, 3.72) months (**Figure 2**).

Based on WAC pricing the total cost of therapy was noted to be $466,040 and average cost per dose was $10,592. Many patients received just one dose of ICI therapy during admission. Six patients received two doses of ICI during admission, three patients received three doses during admission, and one patient received four doses (two cycles of nivolumab and ipilmumab).

### Pretreatment clinical and blood biomarkers were correlated with poor clinical outcomes to ICIs

**Figure 3:**
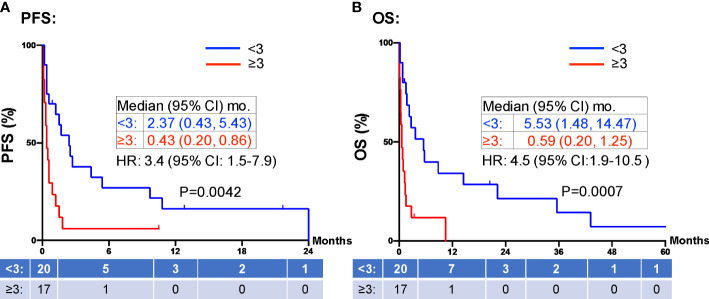
PFS and OS stratified by comorbidities. Patients with greater than 3 comorbid conditions (≥3 red) were associated with shorter PFS **(A)** and OS **(B)** compared to those patients with fewer than 3 comorbid conditions (<3, blue). Groups were compared using the log-rank test. Tick marks indicate censored data. P<0.05 for statistical significance. PFS, progression free survival; OS, overall survival.

**Figure 4:**
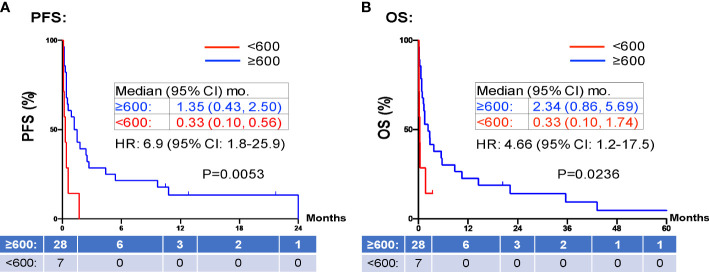
PFS and OS stratified by ALC. Patients with high ALCs (≥ 600 K/mm^3^ shown in red) prior to inpatient treatment was associated with shorter PFS **(A)** and OS **(B)** compared to those patients with low ALCs (<600 K/mm^3^ shown in blue). Groups were compared using the log-rank test. Tick marks indicate censored data. P<0.05 for statistical significance. ALCs, absolute lymphocyte counts; dNLR, derived-neutrophil-to-lymphocyte ratio, PFS, progression free survival; OS, overall survival.

**Figure 5:**
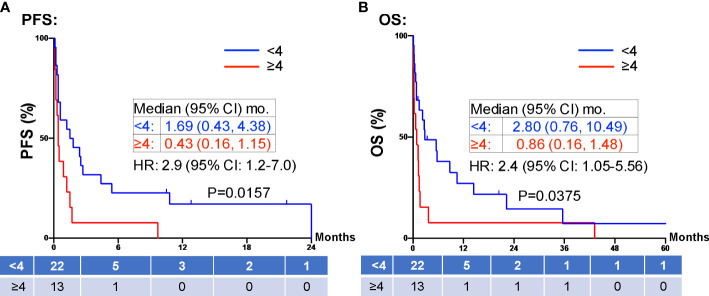
PFS and OS stratified by dNLR. Patients with high dNLR (≥4, red) at prior to inpatient treatment was associated with shorter PFS **(A)** and OS **(B)** compared to those patients with low dNLR (<4, blue). Groups were compared using the log-rank test. Tick marks indicate censored data. P<0.05 for statistical significance. ALCs, absolute lymphocyte counts; dNLR, derived-neutrophil-to-lymphocyte ratio. PFS, progression free survival; OS, overall survival.

When evaluating the effect of comorbid burden on prognosis, subjects with 0 to 2 comorbidities had a better prognosis (both PFS and OS) than those with 3 or greater comorbid conditions (2.4 vs. 0.4 months; HR 3.4, 95% CI 1.5-7.9, *p*=0.0029 and 5.5 vs. 0.6 months; HR 4.5, 95% CI 1.9-10.5, *p*=0.0006) (**Figure 3**). Two of the 37 identified cases were excluded from the ALC analysis due to incomplete blood count records at the time of admission. Evaluation of the remaining 35 patients demonstrated that pre-treatment ALC values of less than 600 cells/µL were associated with poor PFS (0.33 vs. 1.35 months; HR 6.9, 95% CI 10.8-25.9, *p*=0.0053) and poor OS (0.33 vs. 2.34 months; HR 4.66, 95% CI 1.2-17.5, *p*=0.0236) (**Figure 4**). Furthermore, patients with ALC <600 were less likely to receive a subsequent ICI dose than their counterparts (28.6% vs 36.4%). **Table 2** summarizes observed hospitalization duration, ECOG assessments, and ALC characteristics with survival data. Furthermore, when compared to those patients with high dNLR, a low dNLR (defined as less than 4) was associated with a better median PFS (1.6 vs. 0.4 months; HR 2.9, 95% CI 1.2-7.0, *p*=0.0157) and OS (2.8 vs. 0.9 months; HR 2.4, 95% CI 1.05-5.56, *p*=0.0375), respectively (**Figure 5**). Notably, grade 3 and 4 immune mediated adverse events are summarized in **Table 3**, of which two patients required upgrading care to intensive care unit.

**Table 2:** Patient clinical and laboratory treatment characteristics.

No. Patients: N (%)	N=37
**Average comorbidities:**	2.24
**Average PS (ECOG):**	
1	18 (48.6%)
2	8 (21.6%)
3	10 (27.0%)
4	1 (2.7%)
**Duration of admission (Days)**	24.2 (23.1)
**Median ALC (** ± **SD) (N=36)**	1.2, 1.32 (1.08)
**ALCs:**	
ALCs ≥ 0.6	28
ALCs < 0.6	7
**Patients who received a follow-up ICI dose**	
ALCs ≥ 0.6	10 (35.7%)
ALCs < 0.6	0 (0%)
**Median (95% CI) PFS (N=35, in months)**	0.86 months
≥ 0.6 (N= 28)	1.35 (0.43, 2.50)
< 0.6 (N= 7)	0.33 (0.10, 0.56)

PS, performance status; ECOG, Eastern cooperative oncology group; ALCs, absolute lymphocyte counts; SD, standard deviation; ICI, immune checkpoint inhibitor; PFS, progression free survival; CI, confidence interval.

**Table 3:** Immune related severe adverse events (grade 3 or 4).

Severe Adverse Events	Number of cases	Cancer type	Type of ICI treatment
Acute interstitial nephritis	1 (2.7%)	Genitourinary carcinoma	Pembrolizumab
Acute kidney injury	2 (5.4%)	Adenocarcinoma of lung	Pembrolizumab
Acute kidney injury	1 (2.7%)	Adenocarcinoma of prostate	Pembrolizumab
Pneumonitis	1 (2.7%)	Squamous cell carcinoma of lung	Pembrolizumab
Hypersensitivity reaction	1 (2.7%)	Adenocarcinoma of lung	Pembrolizumab

## Discussion

Our institutional review of patients who received ICI therapy while admitted revealed inpatient ICI treatment is associated with a poor clinical prognosis and high cost of therapy. In our study, the most common type of cancer based on site of origin was lung, which included both small cell and non-small cell lung cancer (NSCLC). As seen in the IMPOWER133 study, patients with extensive stage small cell lung cancer who received first line atezolizumab plus chemotherapy demonstrated a median OS of 12.3 months (24). Similarly, the KEYNOTE-189 investigators showed that in patients with metastatic NSCLC who were treated with first line pembrolizumab plus chemotherapy achieved a median OS of 12 months, and median PFS of 8.8 months (25). The results observed in our sample population (median PFS of 0.86 months and median OS of 1.55 months) are significantly worse than would be customarily anticipated.

Hospitalized ICI therapy candidates represent a minority of patients with a dearth of information available to guide clinical practice. The use of chemotherapy in patients who are admitted and in those with poor functional status has been observed and generally accepted as being associated with worse clinical outcomes (14, 26). Performance status as determined by the use of ECOG or similar physical status assessment is an integral part of pretreatment evaluation for the survival of outpatients with advanced cancer (27). Similarly, studies evaluating use of ICI therapy in NSCLC patients with poor functional status has demonstrated similarly worse prognosis than functionally “fit” patients (28–30). Because of the comparatively more tolerable toxicity profile, and aforementioned lack of clinical data, the role of ICI treatment while hospitalized is less clear. Our investigation led to the identification of several predictive factors that can assist decision making in this population. As shown in **Figure 3**, the presence of increasing comorbidities (greater than three comorbid conditions) was associated with a statistically significant worse survival (PFS and OS) compared to those with a lower number of chronic illnesses.

The safety and efficacy of ICI therapy has been demonstrated in numerous clinical trials for a variety of cancers (31–33). However, many patients were excluded from these trials due to concurrent significant medical comorbidities. In practice, patients are given these therapies despite being excluded from the seminal trials. There are few studies to date that study the safety and efficacy of ICI therapy in patients with significant medical comorbid burden. A retrospective review of outpatients who received ICI therapy who had major organ (renal, cardiac, or hepatic) dysfunction showed that these patients did not experience a higher incidence of irAE’s and had durable response rates (34). However, this is in the setting of preserved performance status and functional reserve. In contrast, hospitalized patients with comorbidities often have a reduced physical status simply by definition of being hospitalized. Our study demonstrates that a high comorbid burden (i.e., major organ dysfunction) and a loss of functional reserve (by being hospitalized) is associated with poorer clinical outcomes (**Figure 3**). Due to the ease of use and our findings herein, the continued deployment of routine functional status assessments, assessment of comorbid conditions, and comprehensive medical history taking remain important tools in determining treatment candidacy.

PD-1 and PD-L1 inhibitors up-regulate T-cell mediated anti-tumor activity, and thus rely on the presence of functional lymphocytes (35). Therefore, it is reasonable to hypothesize that a low pre-treatment ALC is associated with a poor response to ICI therapy. Our study confirms this finding in line with previous reports (36, 37), showing pre-treatment ALC <600 cells/µL were associated with poor PFS (0.33 vs. 1.35 months; HR 6.9, 95% CI 10.8-25.9, p=0.0053) and poor OS (0.33 vs. 2.34 months; HR 4.66, 95% CI 1.2-17.5, p=0.0236) (**Figure 4**). Furthermore, patients with ALC <600 cells/µL were less likely to receive a subsequent ICI dose than their counterparts (28.6% vs 36.4%). dNLR is thought to represent a systemic inflammatory state. Inflammation is one mechanism of immune resistance which can lead to activation of tumor growth signaling pathways (38). In recent years, dNLR has been used as a novel biomarker to predict response to immunotherapy in various cancers including NSCLC, melanoma and head and neck cancers (39, 40). It has been shown that a high pre-treatment dNLR (indicating a high inflammatory state) is associated with poor OS and PFS in a variety of cancers (37, 39–42). For instance, Bongiovanni et al, observed a positive association between OS and a NLR ≤ 5 (42). Moreover, NLR < 4 at week 8 of treatment is associated with objective response to treatment (43, 44). In our study, we confirmed pre-treatment dNLR <4 was associated with good median PFS (1.6 vs. 0.4 months; HR 2.9, 95% CI 1.2-7.0, p=0.0157) and OS (2.8 vs. 0.9 months; HR 2.4, 95% CI 1.05-5.56, p=0.0375) (**Figure 5**). The use of both dNLR and ALC may be useful in assessing a patient’s “immune fitness” prior to the initiation of immune checkpoint therapy and may be helpful in predicting response to therapy. Nevertheless, the median PFS of 1.35 months and median OS of 2.34 months in our study patients with pre-treatment ALC ≥600 cells/µL, and the median PFS of 1.6 months and median OS of 2.8 months in patients with dNLR <4, respectively, argues against the use of ICI in hospitalized cancer patients. For hospitalized cancer patients, systemic chemotherapy is frequently used to elicit rapid reduction of tumor burden and symptomatic improvement in patients with chemotherapy-naïve or -sensitive solid tumors (45). However, compared to outpatient chemotherapy, urgent inpatient chemotherapy was associated with higher cost, increased mortality, worse clinical response, and higher mortality rates. These hospitalized cancer patients also had higher comorbidities, longer length of stay, higher discharge rates to skilled nursing, and increased inpatient mortality (46). Despite the hope that ICI might induce significant, durable tumor response with favorable toxicity profile, our study does not support inpatient use of ICI due to the low clinical response. Due to the high cost, ICI uses in hospitalized patients is not cost effective compared to chemotherapy.

Although elderly (≥65 years old) patients consist of over 50% of cancer patients, they are underrepresented in the clinical trials leading to the FDA approval of ICI trials. Currently data suggest age does not significantly affect the tolerability and clinical response to ICI monotherapy (47). However, aging is associated with “immunosenescence”, which includes dysregulation of both cellular and humoral immunity; and is associated with lymphocyte depletion, fewer CD4+ and CD8+ T cells, decreased diversity of regulatory and memory T cells, defective DNA repair response pathway, and metabolic changes. In addition, aging is associated with “inflammaging”, which has an overall increased pro-inflammatory state. All these factors were associated with decreased response to ICI therapy (47). In our study, the mean age was 53.5 years, and 13 (35%) of cancer patients were ≥65 years old. Although ALC was lower in elderly patients compared to younger pts (900 vs 1200/µL), dNLR was lower in elderly patients compared to younger patients (2.9 vs 3.2). It is likely that our hospitalized patients had more inflammatory changes from acute factors other than aging. Further studies are needed to evaluate the role of these easily accessible blood biomarkers to evaluate the immune fitness in predicting prognosis and ICI response in elderly patients.

Lastly, ICI therapy can incur significant costs to both the patient and healthcare system. This is particularly true in the inpatient setting where the cost is not reimbursable and valuable discounts, such as utilization of a 340b pharmacy program, are not applicable. While qualification for 340b (or similar) programs does require certain regulatory and institutional standards to be met, outpatient payments in general treat ICI treatment as a per line charge. This is in stark contrast to inpatient payments that are almost universally bundled into a daily charge without specific treatment carved out in the billing. In this study, we show that the high overall cost and cost per dose does not necessarily lead to significant overall survival. In addition, when taking into account toxicities associated with ICI therapy, the cost can increase exponentially (for instance, one patient in our review developed pneumonitis after ICI administration necessitating intensive care unit admission). Future studies include assessing data from the Surveillance, Epidemiology, and Results (SEER) program database to compare costs nationally and between institutions.

While our study offers new insights into clarifying hospitalized patients have poor clinical outcomes to ICI treatment, significant limitations exist. Most notably our review represents only a single institution that is geographically confined to northern California. Furthermore, we were only able to identify small sample population over the course of 5 years that received ICI treatment while hospitalized. As a result of this small sample size our pooled population represents a diverse group of malignancies with different histologic groups. Additionally, by design our study only offers observational data. As it is based on institutional pharmacy review, further investigation *via* retrospective cohort study, or prospectively with the inclusion of a control group that would allow recruitment of diverse populations which our study was unable to by nature of being observational, would offer superior information to draw conclusions from.

## Conclusion

The results of our investigation suggest that in general ICI therapy offered to hospitalized patients should be provided cautiously. Clinical assessment tools such performance status, assessment of comorbid conditions, and thorough history taking continue to offer benefit in guiding treatment decision making. Furthermore, utilization of simple blood tests for pre-treatment ALC and dNLR may help to assess the “immune fitness” and identify appropriate candidates for inpatient therapy. Further studies are needed to assess the “immune fitness” of cancer patients receiving ICI treatment, especially in the inpatient setting.

## Data availability statement

The original contributions presented in the study are included in the article/**Supplementary Material**. Further inquiries can be directed to the corresponding author.

## Ethics statement

The studies involving human participants were reviewed and approved by University of California, Davis Protocol No. 937274). Written informed consent for participation was not required for this study in accordance with the national legislation and the institutional requirements.

## Author contributions

TL contributed to the conception and design of the study. All authors contributed to the acquisition, analysis, or interpretation of data. RY, HP, and TL drafted and revised the manuscript. All authors contributed to the article and approved the submitted version.

## Funding

This work was supported by the Personalized Cancer Therapy Gift Fund (TL). The Biostatistics Shared Resource (SC) was supported by the UC Davis Comprehensive Cancer Center Support Grant (CCSG) awarded by the National Cancer Institute (NCI P30CA093373).

## Conflict of interest

The authors declare that the research was conducted in the absence of any commercial or financial relationships that could be construed as a potential conflict of interest.

## Publisher’s note

All claims expressed in this article are solely those of the authors and do not necessarily represent those of their affiliated organizations, or those of the publisher, the editors and the reviewers. Any product that may be evaluated in this article, or claim that may be made by its manufacturer, is not guaranteed or endorsed by the publisher.
